# The 5-C model of digital health accompaniment: empowering women through a gestational diabetes self-care intervention in Vietnam

**DOI:** 10.3389/fcdhc.2026.1738433

**Published:** 2026-05-04

**Authors:** Tine M. Gammeltoft, Thị Kim Dung Vũ, Ngọc-Anh Thị Đạng, Thị Minh Phương Nguyễn, Ái T. Nguyễn, Ib C. Bygbjerg

**Affiliations:** 1 Department of Anthropology, University of Copenhagen, Copenhagen, Denmark; 2 Faculty of Public Health, Thai Binh University of Medicine and Pharmacy, Hưng Yên, Vietnam; 3 Department of Public Health, Global Health Section, University of Copenhagen, Copenhagen, Denmark

**Keywords:** accompaniment, digital health, empowerment, gestational diabetes, pregnancy, self-care, Vietnam

## Abstract

**Background:**

Globally, gestational diabetes mellitus (GDM) is a growing health problem. When diagnosed with GDM, women often lack adequate health systems and social support, finding themselves alone at the frontier of epidemiological change. This ethnographic study investigated experiences of GDM and self-care practices among women in Vietnam, with a particular focus on the significance of an online intervention for women’s self-care agency.

**Methods:**

Ethnographic data from in-depth interviews with 42 women with GDM living in the Red River delta of northern Vietnam were analyzed. The research was conducted in two phases. In the first phase, women with GDM received standard care, while women in the second phase participated in a digital pregnancy care intervention combining peer- and professional support. Ethnographic data from both phases were systematically coded, analyzed, and compared.

**Results:**

The study found that the intervention achieved its impact through *accompaniment*, supporting GDM self-care through presence and human connection. While women receiving standard care often found themselves in a stressful impasse between Internet-derived information emphasizing the dangers of GDM and family members urging them to ignore the condition, women involved in the digital intervention mobilized knowledge and support attained through the intervention to actively seek assistance from family members and carve out spaces for self-care. Women used the intervention to leverage family love. To capture the factors that made this intervention a source of accompaniment, we developed the 5-C model of digital health accompaniment, highlighting how *co-creation, combined knowledge, community spirit, continued presence*, and *commitment over time* facilitated health care accompaniment.

**Conclusions:**

For many women worldwide, pregnancy is an emotionally intense period marked by high stakes and anxious expectations. When GDM is diagnosed, uncertainties tend to intensify. In this context, our findings suggest that digital interventions can serve not merely as tools for health education or monitoring, but as infrastructures of accompaniment that strengthen women’s self-care, while also enabling them to mobilize family members’ attention and support. We argue that such accompaniment represents an important source of reproductive empowerment that warrants greater attention in research, policy, and practice, holding potential to contribute to health system strengthening.

**Clinical trial registration:**

https://anthropology.ku.dk/research/research-projects/current-projects/_living-together-with-chronic-disease-part-ii/, identifier NCT05744856.

## Introduction

Gestational diabetes mellitus (GDM), an abnormal glucose metabolism diagnosed during pregnancy, can have serious adverse health consequences for pregnant women and the children they expect (1, 2). The prevalence of GDM is currently rising rapidly worldwide, and the condition is now one of the most common complications of pregnancy (3). This confronts increasing numbers of pregnant women with a diagnosis that places new demands and expectations on them, introducing new forms of pressure, anxiety, and uncertainty (4–6). Existing research shows that many women react with distress and confusion when GDM is diagnosed (7), and that healthcare systems often provide insufficient support, particularly in settings where healthcare resources are limited (8, 9). Research has shown that appropriate GDM self-care can significantly enhance women’s well-being and the outcomes of their pregnancies (10). Existing research in this field, however, has focused mainly on the quantitative connections between self-care and pregnancy outcomes, while qualitative understanding of the underlying socio-cultural processes is limited. To enhance the impact of self-care interventions and strengthen women’s self-care capacities, there is a need for a deeper understanding of the local social dynamics that shape GDM self-care in specific social settings (11).

This ethnographic study was conducted in Vietnam, a rapidly developing middle-income country that currently sees rising rates of non-communicable diseases (NCDs) (12–15). This includes all forms of diabetes, including GDM (3, 16). In Vietnam, as in many other low- and middle-income countries, these epidemiological developments pose serious challenges to the national healthcare system, which remains primarily focused on curative care and prevention of infectious diseases, with less attention paid to the management of NCDs (17–19). The capacity to diagnose GDM is new in most localities in the country, with oral glucose tolerance testing (OGTT) offered on an *ad-hoc* basis and only against out-of-pocket payment, and with limited health provider support when GDM is diagnosed (20).

Taking an ethnographic approach, this study investigated how women in northern Vietnam experience and respond to a diagnosis of GDM, focusing particularly on the role of informal support in everyday self-care. Following the World Health Organization (WHO), we define self-care as “the ability of individuals, families and communities to promote health, prevent disease, maintain health, and cope with illness and disability with or without the support of a health worker” (21), approaching self-care as an important precondition for public health advancement. The research was informed by the WHO’s conceptual framework on self-care interventions, with particular attention to the enabling environment that underpins self-care practices (21). Drawing on previous research on self-care for reproductive health, we attended particularly to reproductive empowerment, basing the study on the assumption that there is a mutually reinforcing relation between self-care and empowerment (22). The research, therefore, investigated not merely the self-care practices that women with GDM undertake, but also their self-care agency, as they perceived and experienced it themselves. In focus was, therefore, the women’s *capacities* to act, and the social conditions that enhanced or restricted such capacities. Whereas most studies on self-care in health focus on individual knowledge and practices, the present study took a family perspective, recognizing that most pregnant women in northern Vietnam lead their daily lives within close interdependent relationships with family members and kin (23, 24).

This article presents one of our most striking ethnographic findings: experiences of accompaniment were at the heart of self-care strengthening. Women who felt accompanied – by healthcare professionals, peers, or family members – expressed a markedly stronger sense of being in control of their antenatal care and more robust capacities to practice GDM self-care than women who described feeling lonely and unsupported. In this article, we highlight the social processes through which such health care accompaniment was accomplished. We use the term accompaniment with inspiration from Paul Farmer who defines accompaniment as a standing alongside people in their strivings for health; a stance that exceeds merely technical delivery of health care services: “To accompany someone is to go somewhere with him or her, to break bread together, to be present on a journey with a beginning and an end. There’s an element of mystery, of openness, in accompaniment: I’ll go with you and support you on your journey wherever it leads. I’ll keep you company and share your fate for a while. And by ‘a while,’ I don’t mean a little while. Accompaniment is much more often about sticking with a task until it’s deemed completed by the person or people being accompanied, rather than by the *accompagnateur*” (25). In this perspective, accompaniment is a matter of forming relations of solidarity with others, participating in their care in sympathetic ways. Taking inspiration from Farmer, this article presents an ethnographic exploration of the dynamics of accompaniment in the field of pregnancy and childbearing in northern Vietnam, focusing on the role of an online self-care intervention as a digital companion.

## Materials and methods

This ethnographic study was conducted under the auspices of the larger, interdisciplinary research project titled “Living Together with Chronic Disease: Informal Support for Diabetes Management in Vietnam. Phase II: Gestational Diabetes in Vietnam” (VALID II), which included a digital GDM self-care intervention (hereafter, the VALID II intervention) (26). This article documents how the intervention was taken up and used by pregnant women with GDM and their families, focusing particularly on its impact on the women’s self-care practices. Research findings on clinical outcomes are reported elsewhere (12, Le et al., 2026^1^).

### Study setting

The research was conducted in Thái Bình province (since 2025 renamed Hưng Yên province) in Vietnam’s Red River delta, 120 kilometres southeast of the country’s capital, Hanoi. At the time of the research, Thái Bình had a population of around 2 million people whose livelihoods relied mainly on agricultural production and industrial employment. Like other provinces in Vietnam, the province currently confronts a rapidly rising prevalence of NCDs, including GDM. In 2023-2024, VALID II research found a prevalence of 27.1% among women attending antenatal care in Thái Bình City (16). OGTT testing for GDM has been available in the province since 2018, against out-of-pocket payment. At the time of this research, no systematic counselling or follow-up care was provided when a woman tested positive for GDM at private or public health facilities. Follow-up care was offered on a shifting and *ad-hoc* basis: the care each woman received varied, depending on the knowledge and skills of the healthcare provider and the woman’s situation and relationship with the provider (20).

### Study design and sample

The VALID II project forms the overall frame for this ethnographic study. VALID II includes three main components: an epidemiological study investigating GDM prevalence and GDM self-care among pregnant women attending antenatal care in Thái Bình City; an ethnographic study following women with GDM over time, from the diagnosis until at least 12 months postpartum; and an online intervention aiming to enhance self-care capacities among pregnant women with GDM. Data collection sites for the epidemiological study were two healthcare facilities: a public maternity hospital and a private antenatal care clinic, both located in Thái Bình City. All women attending antenatal care at these two sites in 2023 and 2024 were invited into the study and offered a free OGTT test. In Phase 1, conducted in 2023, women diagnosed with GDM received standard care, while in Phase 2, conducted in 2024, the women were invited to join the intervention. A total of 435 women (233 in 2023 and 202 in 2024) were diagnosed with GDM and enrolled in the study. Of the 435 women with GDM, 42 women took part in the ethnographic study (21 in each phase). All 42 women were married, and they all considered their economic status “medium.” Approximately half of the women lived in nuclear families, while the other half lived in extended families with two or three generations. In Phase 1, nine women were pregnant with their first child, and in Phase 2, six women were pregnant with their first child, while most women had one or more children already. For details regarding the sample, see **Table 1** and **2**.

**Table 1:** Non-intervention sample (2023).

Characteristics		Participants (N = 21)	% of total	Participants in full study (N = 233)	% of total
Age	Mean (range)	32.3 (20-47)		30.5 (19-47)	
Education	Primary school	0	0	1	0.4
	Secondary school	2	9.5	27	11.6
	High school	8	38.1	55	23.6
	Intermediate/college/university/postgraduate	11	52.4	150	64.4
Occupation	Factory worker	7	33.3	67	28.8
	Teacher/office worker/Health staff	9	42.9	115	49.3
	Self-employed	5	23.8	21	9.0
	Student/not working	0	0	30	12.9
Family economic status (self-assessed)	Poor/near poor	0	0	3	1.3
	Medium	20	95.2	226	97.0
	Wealthy	1	4.8	4	1.7
Marital status	Married	21	100	230	98.7
	Unmarried/divorced	0	0	3	1.3
Household	Two-generational	9	42.9	106	45.5
	Three-generational	10	47.6	123	52.8
	Four-generational	2	9.5	4	1.7
Number of living children	0	9	42.8	82	35.2
	1	3	14.3	84	36.1
	2	7	33.3	55	23.6
	3	1	4.8	10	4.3
	4	1	4.8	2	0.9

**Table 2:** Intervention sample (2024).

Characteristics		Participants (N = 21)	% of total	Participants in full study (N = 202)	% of total
Age	Mean (range)	32.3 (25–44)	–	30.2 (19-46)	–
Education	Primary school	0	0	0	0
	Secondary school	2	9.5	14	6.9
	High school	5	23.8	56	27.7
	Intermediate/college/ university/postgraduate	14	66.7	132	65.4
Occupation	Factory worker	7	33.3	50	24.8
	Teacher/office worker/ health staff	8	38.1	62	30.7
	Self-employed	4	19.1	46	22.7
	Student/not working	2	9.5	40	19.8
Family economic status (self-assessed)	Poor/near poor	1	4.8	2	1.0
	Medium	20	95.2	200	99.0
	Wealthy	0	0	0	0
Marital status	Married	21	100	199	98.5
	Unmarried/divorced	0	0	3	1.5
Household	Two-generational	12	57.2	95	47.0
	Three-generational	9	42.8	98	48.5
	Four-generational	0	0	9	4.5
Number of living children	0	6	28.6	75	37.1
	1	5	23.8	76	37.6
	2	9	42.8	44	21.8
	3	1	4.8	7	3.5

#### The intervention

The intervention was designed at a co-creation workshop held in Thái Bình City in August 2023, drawing on insights from the first phase of ethnographic research. Following suggestions from workshop participants, the intervention was developed with an emphasis on digital communication, relying mainly on the Vietnamese communication app Zalo. Zalo is one of the most widely used communication platforms in Vietnam, with nationwide coverage and millions of users. Conducting the intervention digitally was seen by co-creation workshop participants as the most feasible approach due to the flexibility it offered in terms of time and space for participation. At the heart of the intervention were nine peer groups established on Zalo, with around 20 women in each group. The Zalo groups ran from the time of GDM diagnosis until four months postpartum. Each group was supported by a moderator and a health professional who answered the questions from the participants that required professional responses. The health care provider responded only to questions from the women that were of general interest to members of the Zalo group, while women posing more specific or personal questions were encouraged to consult their antenatal care provider.

When joining the Zalo group, the women received a welcome message formulated in a warm and caring tone. This welcome message emphasized the collective nature of the intervention, stressing its non-commercial nature and encouraging the women to share their thoughts and experiences: “We encourage you to share your personal experiences regarding how to live well with GDM, supporting one another to have a joyful and good pregnancy journey” (Chúng tôi khuyến khích chị/em chia sẻ về các kinh nghiệm, trải nghiệm cá nhân về cách sống khỏe mạnh với ĐTĐTK, hỗ trợ lẫn nhau để có được một hành trình mang thai vui vẻ và tốt đẹp)!. The two Vietnamese terms for “experience” used in this message point to practical knowledge gained over time (kinh nghiệm) as well as more personal, emotional experience of going through events (trải nghiệm). Depicting pregnancy metaphorically as a journey, the message stressed the importance of togetherness and mutual support in this passage into motherhood. The welcome message ended with these words: “Let’s hope we can support one another to have a safe and healthy pregnancy, successfully making the crossing (vượt cạn thành công).”

Along with the welcome message, the women received a set of eight digital educational sheets providing insights and advice on how to live with GDM. Each sheet was accompanied by a video in which a health professional explained the contents of the information sheet. These educational materials were developed based on information needs expressed by women with GDM in Phase 1. The topics covered included: basic information about GDM; nutrition and diet; exercise/physical activity; blood sugar measurement at home; mental health; family support; and childbirth and postpartum care. The digital information leaflets were supplemented by bi-monthly Zoom meetings held across all Zalo groups. The Zoom meetings were guided by a health care provider who gave a short introductory talk on a GDM-related topic, followed by a Q&A session. In the postpartum period, the women received an additional information leaflet on infant feeding. The 202 women diagnosed with GDM in the project’s second phase all accepted the invitation to take part in the intervention; an uptake rate that testifies to the considerable needs for GDM information and support in this population group. The intervention ran from March 2024 to June 2025.

### Data collection and analysis: the extended case study

The key ethnographic research method was the extended case study. This method aims to enable researchers to gain a deeper understanding of a social phenomenon through the detailed investigation of specific cases (27). The extended case studies included participant observation, in-depth ethnographic interviews, and photographs taken during field visits. The ethnographic interviews were based on an open-ended interview guideline that was used flexibly, addressing each woman’s specific concerns. To better understand each family’s situation, the researchers visited the women in their homes, spending two to three hours on each visit. Depending on each woman’s preferences, other family members, such as the woman’s husband, mother, or mother-in-law, also took part in the conversations. We visited the women at home at least once during their pregnancy and at least once during their maternity leave, while also visiting mother and child at the hospital shortly after the delivery. These repeated visits provided insights into the women’s daily lives, while also fostering rapport between the researchers and research participants. It was our impression that these sustained relationships over time strengthened the women’s trust in the researchers, increasing the validity of the research by enhancing the women’s openness around difficult and culturally sensitive themes, such as emotional frustration or experiences of limited family support.

The main topics covered in the ethnographic interviews were family situation and pregnancy experience; the GDM diagnosis and perceptions of GDM; GDM self-care, including support from family members, friends, and colleagues; expectations regarding childbirth and the postpartum period; and thoughts about the future. The interviews with women in the second phase also included open-ended questions about their experiences with the digital intervention. All ethnographic interviews were voice recorded, transcribed, and systematically coded using a thematic list of codes. Ethnographic fieldnotes, recordings, photos, and transcriptions were safely stored on an online platform. All personal names used in this article are pseudonyms.

## Results

Our thematic analysis of the ethnographic materials focused on the women’s social and emotional experiences of the GDM diagnosis, with particular attention to their self-care practices. In women’s accounts of their GDM experiences, two themes emerged as particularly significant: access to information about GDM and psychosocial support from family/household members. To illustrate the importance of these two themes for the women’s pregnancy experiences and GDM self-care, we present four ethnographic vignettes drawn from our extended case study (28). Two vignettes (vignettes 1 and 3) are based on ethnographic material from the non-intervention group, and two (vignettes 2 and 4) on ethnographic material from the intervention group. We have selected these four cases, as they illustrate with particular poignancy the importance of accompaniment during a pregnancy affected by GDM.

### Quests for knowledge and information: the role of accompaniment

#### Vignette 1: Thảo (41 years old, pregnant with her third child)

The day she was diagnosed with GDM, on March 18, 2023, Thảo went straight home and googled “đái tháo đường thai kỳ” (gestational diabetes) on her smartphone. At the hospital, staff was busy, and the doctor had simply handed her the positive test result without any further explanations. When she reached her home in the early afternoon, her daughters were still at school, and the house was quiet. Sitting on her living room sofa, reading through the Internet sites that emerged on her screen, Thảo felt frightened. “Gestational diabetes can cause serious health complications for mother and child”, she read, her heart racing. “Gestational diabetes poses several risks to the baby, such as excessive growth … premature birth … severe breathing difficulties … low blood sugar … birth defects … death shortly after birth … risk of obesity and type 2 diabetes in adulthood … stillbirth…”. Feeling dizzy, Thảo stopped reading, took a photo of the text describing GDM complications, and sent it to her husband via Messenger. Soon after, he called her, saying one word only: “Kệ”, “ignore it.” With tears in her eyes, Thảo described how her husband disregarded her GDM, saying simply, “our two children are fine, so this one will be fine too.” As she continued her Internet searches, reading about GDM, Thảo’s feelings of loneliness deepened. “I wish I had received some kind of guidance”, she said:

“Guidance that explained how I can understand my blood sugar levels, what my diet should be like, and how I should exercise. But they (health providers) did not give me any advice. I had to figure out everything on my own. … I wish they would pay more attention and give me more concrete guidance (quan tâm hơn và có cái hướng dẫn cụ thể hơn). That’s my wish. As a patient, when you come to the hospital, you want to be able to trust the doctor, to receive authoritative information. Then you feel more reassured. Finding information on one’s own … you don’t know what you can trust and what’s unreliable. I prefer authoritative guidance (*cái chính thống*).”

#### Vignette 2: Nhi (30 years old, pregnant with her third child)

Nhi had her GDM test at a private antenatal care clinic in her 24th week of pregnancy. When the doctor told her she had tested positive, Nhi was puzzled. She had not expected this at all. “There were no problems in my first two pregnancies”, she told us, “And I don’t usually eat sweet things or drink sugary beverages. So why did this happen? At the clinic, they told me only that I should cut down on rice and sweet fruits in my diet. They did not give me any other information.” “I used to eat two bowls of rice at each meal”, Nhi said, “now I eat only two spoons. But hearing I had GDM, I felt so scared and uncertain (băn khoăn) regarding what to eat and what not. I think the most important thing is one’s diet.” When she was invited to join the Zalo group, Nhi accepted without hesitation. Eager to learn more about GDM, she carefully studied the dietary advice she received in her Zalo group and followed the recommendations, increasing her intake of vegetables and greens substantially: “When I ate like that, I felt better. I also felt I wasn’t so tired anymore. I heard people say diabetes makes you tired, or maybe it was just psychological, I don’t know—but I did feel tired. But when I followed the diet … it’s like my spirits (tinh thần), you know, they really improved.”

When she had trouble controlling her blood sugar and felt confused about her diet, Nhi asked for diet advice in her Zalo group. “When I asked, the doctor responded immediately”, she said. “She seemed very committed (nhiệt tình).” When learning she had tested positive for GDM, Nhi talked to her husband, mother, and friends. She and her husband share their house and daily life with her 70-year-old mother, while her husband’s parents live in a neighboring district. “My mum is 70, but she is much stronger than I am”, Nhi laughs, “and she supports me a lot, especially with food. I told her, ‘I have diabetes, so you must cook more vegetables for me, I cannot eat a lot of fatty meat.’ And so she cooks food that’s good for me.”

Like Thảo and Nhi, practically all women in this study expressed profound disorientation and doubt when first informed that they had tested positive for GDM. Many women had children already, and as their previous pregnancies had been unproblematic, they had not expected problems to occur. To most, therefore, the GDM diagnosis came as a surprise and a shock. As GDM testing had only recently been introduced, this was a new health problem, and most women had only vague knowledge of its nature and potential implications. At the health care facility where they were diagnosed, they received only very cursory information about GDM from health care staff, with no or limited opportunity to ask questions. Instead of offering counselling, antenatal care staff would often simply hand the woman the telephone number of a physician at the endocrinology department at a nearby general hospital. Few women, however, had the courage and energy to contact this physician, explaining that they felt too “shy” (ngại) to reach out to him and/or that they did not want to bother other people. In a social setting where medical hierarchies are steep, making an unsolicited phone call to a physician one does not know is not easy. As Thanh explained, “At the clinic, I had to wait and wait, and then eventually they did not explain anything. They just handed me a phone number and told me to connect with this doctor. So I gave up … I don’t want to bother other people.” When we asked Thanh what kind of GDM care she would have liked to receive, she replied: “I wish the doctors would take the initiative themselves and offer me some information, right there, at the antenatal care clinic. Instead of sending me home and expecting me to call another doctor myself. This feels very awkward. I felt very uncomfortable with that.”

While the reactions of uncertainty and disorientation were shared across our sample, there were significant differences between the intervention and the non-intervention groups when the first shock and surprise had settled. In the non-intervention group, as illustrated by Thảo’s case, the momentary confusion at the time of diagnosis seemed to become permanent, continuing throughout the pregnancy. Lacking information and support from a health care provider, nearly all women turned to the Internet for information. Here, they found “TikTok doctors” providing GDM information while also selling pregnancy merchandise for commercial companies; Facebook groups for pregnant women moderated by physicians who earned extra income by providing counselling services; websites from hospitals and commercial companies offering information about GDM; and scattered news items about the dangers posed by GDM. Overall, this was an information jungle where many women felt lost in the wilderness of contradictory and confusing healthcare messages. They all seemed to be acutely aware that the Internet was a marketplace where numerous actors were seeking to gain an income, and they felt unsure as to which information to trust and which not. Forty-one-year-old An, for instance said, “I searched by myself on the Internet. I just typed it in and looked it up. But there are so many different sites … I wish that when I go for a check-up, the doctor would tell me what to do. Then I would follow that advice.” In many cases, this sorting through Internet-derived information was a quest for knowledge that the women undertook on their own. In some cases, their family members would react like Thảo’s husband, telling them that their GDM was not a real problem and urging them to disregard the diagnosis; in other cases, they felt that family members, particularly of the older generation, did not understand this kind of reproductive problem and were not able to offer advice.

In the intervention group, the women’s accounts were markedly different. Although feeling disoriented and alarmed at first, the women seemed to soon regain mental balance, appreciating the advice and information they received from their Zalo group. As Nhi put it, “It’s good to have someone guiding me in what I should do (chỉ rằng mình cần phải làm những cái gì), someone who is attentive to how I eat (sát sao là ăn uống như thế nào).” Both of the Vietnamese terms for guidance and attentiveness that Nhi uses here - chỉ rằng and sát sao – evoke a feeling of being supported through another person’s involvement in one’s life through active instruction and close attention. Her words evoke a world in which we are not alone as human beings but closely connected; a world in which cultural ideals of compassionate intervention prevail. Similar sentiments were expressed by many other women taking part in the intervention; there was a general appreciation of GDM information being delivered in this immediate, responsive, and attentive way. The Zalo doctors were characterized with terms such as nhiệt tình (warm, enthusiastic, committed) and năng nổ (being energetic, in a way that contributes positively to communal life). The women’s accounts indicated that what was important to them was not just the GDM information as such, in a more technical sense, but also the compassionate and caring way in which it was given, and the community spirit that this generated in the group. In contrast to the women in the non-intervention group who felt isolated and alone as they searched for GDM knowledge, the intervention participants expressed a strong sense of being taken by the hand in their quest for knowledge. In this context, the temporal dynamics of the Zalo interactions were important. Like Nhi, many appreciated the immediate replies they received when they posed questions in the groups. They felt there was someone at the other end, caring about them, and being consistently present. As one woman said, “I feel that whenever any of us felt uncertain about something, we got immediate feedback. The expert responded immediately. Whenever we needed something, she responded.”

Women in the intervention group also stressed the reliability of the information they received from the leaflets, educational videos, and Q&A’s shared through their Zalo group. In contrast to women in the non-intervention group who felt lost in a jungle of potentially unreliable information, they trusted the information they received, finding it “scientific” (khoa học) and authoritative because it came from a medical university. Some women explicitly contrasted “outside information” (kiến thức bên ngoài) with the information they received within their group, relating how “outside” information made them feel frightened (hoang mang) and worried (lo lắng), in contrast to the trust evoked by the reliable and reassuring information they received within their group. As one intervention participant put it, “On the Internet, there are so many sources of information. One person says something is okay, another says it is not. So it’s really hard for me to adjust my diet and daily routine.”

This creation of an “inside” space, a community in which one could feel safe, was supported by the Zalo group moderator who encouraged the women to share their experiences. A typical moderator remark was, “Thank you for sharing. Please, sisters, do share with (name)”; or “Hey, sisters, I encourage you all to share your experiences with (name)”. This encouragement to share appeared to foster a sense of community, making the women feel part of something larger, rather than, like women in the non-intervention group, alone and abandoned with their GDM and the questions it raised. Several women said that although they did not themselves pose questions in the group, they learnt from other women’s inquiries and the answers they received. This combined knowledge-creation, where learning took place in a process involving both peers and professionals, was among the features that seemed to be appreciated the most, giving the women a feeling of being accompanied: of being seen and met in their needs and not journeying alone.

Lastly, many women reported that they shared the information gained in the group with other women in their neighborhood or at their workplace, thus enlarging the community of experience-sharing beyond the circle of participants in the intervention. In Anh’s words, “At the company where I work, we are three who are pregnant, and two have GDM. My colleagues asked me, ‘With this condition, how should one eat?’ I tell them how I eat meat and vegetables, how I limit carbs and sweets, and limit sugary drinks. I also tell them how I divide my meals into smaller portions instead of eating a lot at a time.” Through such community-based sharing, the information provided by the intervention travelled on, reaching larger groups of childbearing women and their families.

### Psychosocial support: the importance of accompaniment

#### Vignette 3: Hoa (40 years old, pregnant with her third child)

“My sister tells me this is normal,” Hoa said with a sigh. She spoke in a quiet tone of resignation as if confronting a large and overwhelming force: “My sister had GDM too, and after she had given birth, everything was back to normal. My mother also says it is normal. Everyone in my family says diabetes in pregnancy is normal. After birth it’s gone, it’s no problem at all, they say…. None of them know anything about GDM, so they say it doesn’t matter. My mother knows nothing about this disease, so she doesn’t worry. She tells me not to worry too … My husband also knows nothing about GDM, he doesn’t understand anything at all. I have to take care of this myself (mình phải tự lo thôi). This is a new disease; few people know about it.”

This was Hoa’s third pregnancy. Working at a textile factory, she lived in a semi-urban area, sharing a house with her husband and two daughters, aged 17 and 11. Her 72-year-old mother-in-law lived nearby and passed by every day, to talk, read with her granddaughters, or see if there was anything she could help with. Yet in a tone of frustration, Hoa told us about how she had to handle her GDM on her own. Whereas her husband had been very supportive during her first two pregnancies, this time she felt completely abandoned. After the diagnosis, she had bought a glucometer to test her blood sugar daily, and she tried to eat a GDM-friendly diet, following advice she found on the Internet. “I take care of this myself,” she said. “My husband does not care. He says, ‘Why don’t you eat anything anymore? No matter what I buy, you don’t eat it. I used to buy the food you like, but now you don’t eat it anymore, so I may as well not buy it.’ He does not know what is good for me and what is not … I try to explain to him that there is a lot of sugar in rice, but he says, ‘Of course there’s not sugar in rice. Rice is not a problem, take it easy. Just eat.’ But how can I just eat when I’m so worried? I’m glad it’s my eldest daughter who cooks our meals. She pays a lot of attention to me.” Apart from her eldest daughter, Hoa said, no one in her family supported her in her efforts to change her lifestyle after the GDM diagnosis. Citing an old adage, she said, “the one who has the disease is the one who carries the worries (mình có bệnh mình phải lo chứ).”

#### Vignette 4: Nga (26 years old, pregnant with her first child)

“Water spinach, mustard greens, cauliflower, cabbage, napa cabbage … I love vegetables”, Nga declared. When she learnt that she had GDM, and the doctor told her to eat lots of vegetables and cut down on rice and fatty foods, Nga imagined that this would not be too difficult. She did not usually eat many sweets and preferred a diet with little meat and without too much salt. But things turned out differently. Lowering her voice, she said, “My parents-in-law … oh heavens. When they stir-fry vegetables, they put so much fat, it’s so greasy that I cannot even eat it … My own parents are different. They cook more carefully. If we’re with them, they pay more attention.”

As a new daughter-in-law in this household, Nga did not feel in a position to make decisions about the family’s daily diet. When she came home from work, her parents-in-law had usually prepared dinner already. Her mother-in-law encouraged her to eat a lot, out of fear that if she was on a diet, the child she was expecting – her mother-in-law’s first grandchild – would not get enough nourishment. GDM is not a problem, her mother-in-law claimed, echoing what we had heard from many other women of her generation: “After birth, it’s gone. Take it easy, it’s not a problem (đẻ xong hết, cũng yên tâm, không vấn đề gì).” Seeing how this pressure to eat burdened his daughter, Nga’s natal father intervened. Living nearby, he had been involved in Nga’s pregnancy from the start, accompanying her for antenatal care visits and studying the information she received in her Zalo group. He went to see Nga’s parents-in-law, sharing the Zalo diet advice with them. This helped a lot, Nga said: now, suddenly, her parents-in-law would take her nutrition needs into account when cooking. This was a great relief, she said. “But,” she continued, “I’m still concerned that my child will grow up to become fat. It’s not good for children’s health to be too fat.” After six months of maternity leave, Nga would have to return to work, and her mother-in-law would then be taking care of her little daughter. “At that time,” she said, again lowering her voice, nearly whispering, “it will be very difficult.”

Striving to adjust their daily habits to a pregnancy with GDM, all women in our study expressed a need for social and emotional support from their families. Like Nga and Nhi, many lived in extended families, sharing a household with their own or their husband’s parents. Women living in nuclear families would often, like Hoa, interact with their parents and parents-in-law daily, relying on other family members’ help and support to nearly the same extent as women who shared a household with the elder generation. Everyday habits were, therefore, of an intergenerational and communal rather than individual nature.

When a GDM diagnosis was made, and they were advised to reduce their intake of white rice and sweet fruits while also eating more vegetables and less fatty foods, most women took this advice very seriously, striving to adjust their diets, while also including time for walking or other forms of exercise in their daily schedule. To implement these changes to daily ways of living, they needed support from family and household members. While this need for support was shared across our sample, there were marked differences between the experiences reported by women in our intervention and non-intervention groups. As illustrated by Hoa’s case, women in the non-intervention group often expressed intense feelings of loneliness, feeling that their family members disregarded and downplayed their GDM. Seeking to comfort and reassure their wife, daughter, or daughter-in-law, family members would often stress the transitory nature of GDM, telling the woman not to worry, as this would soon pass. Twenty-year-old Linh, for instance, told us how her mother-in-law cared for her by encouraging her to eat more and not think about her GDM: “My mother-in-law says, ‘It does not matter, just eat my child’ (thôi cứ ăn đi con ạ, không sao). She constantly encourages me to eat. ‘Do eat, child, it’s not a problem’, she tells me. I think she is right. It’s better just to eat and not think too much. Whatever my mother-in-law says always sounds right to me, so I keep eating without thinking. She tells me to eat to get a fuller figure, so I eat.” While some women, such as Linh, felt reassured by family members’ advice to ignore the condition, most expressed deep frustration. The Internet sources they consulted told them about the health risks that GDM entailed, and yet many were unable to garner their family members’ support for lifestyle changes. Their pregnancy was, they felt, a journey they undertook on their own.

Most women in the intervention group, in contrast, described how they received the social and psychological support they needed, both from peers in their Zalo group and from family members. Many emphasized how important it was to know that they were not alone, as many other women were in the same situation. The women stressed the importance of the Zalo group as a forum where any question could be raised and addressed. In the group, questions such as the following were raised: “Sisters! Can I ask a small question? Is it OK to eat a lot of nuts, like cashew nuts?”; or “Can I drink red apple juice when I have GDM?”; “Sisters, what should I do if I have diarrhea? Can I use digestive enzymes or anything?”; “I am 33 weeks pregnant now. Can I eat pineapple?”; “Can I drink unsweetened milk after giving birth?”; “If my blood pressure is not high, can eclampsia still occur?”; “I often eat soup, such as crab soup, spinach, and squash. Will it affect me? Because I eat soup more often than boiled vegetables. I still eat various fruits as snacks: cucumbers, mangosteen, and rambutans.” As illustrated by these examples, the women’s questions would often focus on diet; on management of common health conditions such as colds or diarrhea; on blood sugar management; and on gynecological/obstetric problems.

The contributions from other Zalo group members were encouraging, it seemed, not just because the women received practical suggestions for living, such as diet or exercise recommendations, but also because other women’s digital presence offered a sense of comfort and accompaniment. Zalo health professionals’ answers to group members’ queries strengthened this feeling of being part of a community; of being accompanied and guided by an expert who had their well-being in mind. In contrast to the women in the non-intervention group who felt they were struggling alone at a reproductive frontier, women in the intervention group expressed feelings of being reassured and helped. Accompaniment thus seemed to enhance the women’s self-care agency: receiving more psychosocial support, they were able to adopt new lifestyles and adapt to the situation in which they found themselves. They had someone supporting them on their journey.

Importantly, in many cases, as in that of Nga, the intervention also served to leverage support from family members. Whereas family members in the non-intervention cohort often tried to support the woman by downplaying her GDM and attaching little importance to it, thus caring by ignoring, most women in the intervention cohort were able to guide family members in how to support them. They did this by mobilizing information gained from the intervention, sharing it with family members and encouraging them to help with diet and exercise. Several women and their husbands, for instance, adopted a new habit of walking together after their evening meal, and in many families, everyday diets were changed to support the pregnant woman’s needs for a GDM friendly diet. In many cases, therefore, the intervention helped to strengthen family solidarity and community spirit. An important precondition for this mobilization of support from family members was the scientific authority of the intervention: the fact that it was run by a trusted local medical university. This authority not only provided credibility in the eyes of the pregnant women themselves but also seemed to boost their self-confidence in sharing information with family members. Women drew, in other words, on the intervention’s scientific authority to influence family dynamics, thus leveraging family love for GDM care. Additional significant features of the intervention’s psychosocial support were its consistent presence in the women’s lives and the commitment over time that it entailed. Whereas consulting a health provider physically requires time and travel across distance, the digital presence of the app made it an almost embodied part of the women’s lives. The Zalo group was there for the women at any time, a source of accompaniment on their journey through the pregnancy, with its constantly changing and evolving needs and questions as the due date neared. The intervention, in short, became an important source of psycho-social support – both directly, through peer feedback and professional guidance, and indirectly, as a tool to mobilize family support.

## Discussion: the 5-C model of digital health accompaniment

In Vietnam, the process of pregnancy and childbirth is often portrayed as a journey. The birth of a child is designated metaphorically as a “crossing” (vượt cạn), hinting at the dangers and hardships involved in bringing a new child into being. The literal meaning of vượt is “to cross”, while cạn refers to shallow waters or low tide. Metaphorically, then, cạn refers to a critical or dangerous moment, while vượt captures the effort needed to steer through that moment. The term vượt cạn is often used to celebrate the courage and bravery of motherhood and the struggles it takes to bring new life into being. Vượt cạn holds strong resonance in water-based Red River delta culture: people here know the difficulties of navigating through shallow and dangerous waterways where boats can easily get stuck, damaged, or overturned. Childbirth is, this metaphor suggests, a life-threatening, liminal, and turbulent passage where dangers are real and survival not guaranteed. Our ethnographic findings show that if giving birth is like navigating a boat through shallow waters, then women need a sense of direction and a feeling of being supported as they undertake this critical passage. Although the birthing woman must do the laboring herself, others can provide support.

These needs for direction and support are not unique to women with GDM in Vietnam. Information and psychosocial support are among the topics highlighted in WHO’s conceptual framework on self-care interventions as significant components of a self-care-supportive social environment. Further, previous studies have found that women with GDM express needs for better information and for sustained social support from family and health care providers (8, 9, 29). This points to the importance of accompaniment when a GDM diagnosis creates turbulence on the pregnancy journey. The term *accompaniment* has roots in the Latin “com” (with, together) and “panis” (bread), with the late Latin term “companionem” referring to “one who shares bread with another.” To accompany thus means to take the other person as one’s companion, with the implicit meaning of sharing meals, and thereby also life, with one another. In Vietnamese, the term for accompaniment is đồng hành; a term that evokes joint traveling: *hành* is “to go” or “to travel”; đồng is “together with” or “same”, i.e., the term refers to collectivity and joint traveling; to being aligned in purpose and direction, with similar connotations of “sharing” as the English term accompaniment.

In global health, the term accompaniment has been used as a guiding ethical and clinical principle by the physician-anthropologist Paul Farmer and the organization Partners in Health that he co-founded in the early 1980s (25, 30). In Farmer’s usage, accompaniment is both a philosophical stance and a model for health program design (31, 32; see also 33). Accompaniment is about providing care based on shared experiences, rather than from a distance. It is about meeting people where they are, understanding their lives and values, and addressing structural barriers to health from this vantage point of shared lives, rather than in a top-down manner. In Vietnam, the term *đồng hành* is used similarly, being employed by hospitals, clinics, and health care providers to describe the way in which they seek to care for their patients. The term is often articulated in official state policy, being used to highlight the close links between the state health sector and the people. As an article published in the Vietnamese Army’s newspaper Quân Đội Nhân Dân in August 2025 declared: “For the past 80 years, since the establishment of the first central health authority (on August 28, 1945) until now, the Vietnamese health sector has accompanied the people through every stage of its history (80 năm qua, từ ngày thành lập Nha Y tế (28-8-1945) đến nay, ngành Y tế Việt Nam đã đồng hành cùng dân tộc trong mỗi chặng đường lịch sử)” (34).

In the pioneering work by Farmer and his collaborators, the term *accompagnateur* is used to designate local community health workers who live and work in physical proximity to the people they serve. In extension of this, our ethnographic findings from Vietnam point to the potential of *digital health actors* as *accompagnateurs*, showing how not only physical but also digital presence can hold important capacity for accompaniment. This finding aligns with recent research in the field of GDM and beyond, which points to the capacities for interhuman care and connection held by digital technologies (35–38).

**Figure 1:**
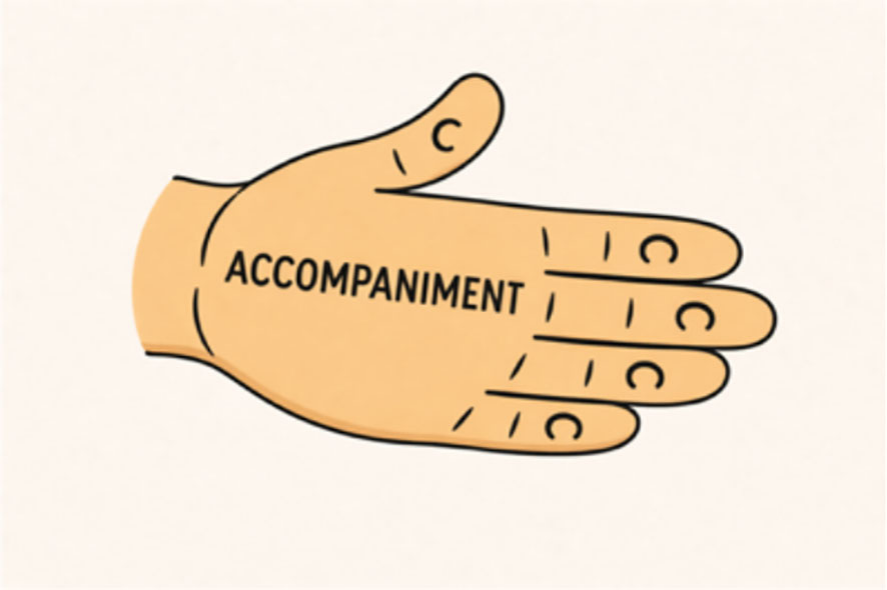
Digital health accompaniment: the 5C-model (AI-generated figure).

To understand how the VALID II intervention became a significant source of accompaniment, we have identified five principles that were key to women’s experiences of being accompanied through a pregnancy with GDM. We designate these five principles: the five-C model of digital health accompaniment. The key characteristics of the five-C model are: Co-created design (developing the intervention *with* participants, not for them); Combined knowledge (combining local knowledge with expert knowledge); Community spirit (creating a “we’re in this together” spirit in contrast to a top-down hierarchical approach); Consistent presence (immediate responses and assistance); and Commitment over time (staying with participants through a transformative life process). As evident from the above summary of our main research results, these five principles emerged from the women’s accounts as significant features of their participation in the intervention. We find the term “5-C model” particularly fitting, as “digital” in the original Latin means “finger”—our term “5-C” thus hints at the five fingers of the hand involved when taking someone by the hand in accompaniment. For a graphic illustration of this, see **Figure 1**. Besides being of use as a mnemonic tool, this figure complements the theoretical analyses by visualizing what it takes to empower women, illustrating how women are accompanied as they take their lives and health into their own hands.

In the context of Vietnam, the 5C model’s spirit of togetherness resonates with longstanding cultural values of solidarity, companionship, and mutual support (23, 39, 40). Accompaniment (đồng hành) was at the heart of socialist-era health care philosophy in Vietnam. As part of Vietnam’s anti-colonial resistance and efforts to build a new, socialist society, a comprehensive primary health care system was established in the 1950s (17, 42), offering preventive health care and basic medical services to citizens. In contrast to the health deprivations that prevailed during colonial rule, equal access to health care for all was placed at the heart of the new, socialist society (41, 42). This basic primary health care system was maintained during the hard years of the Second Indochina War, with healthcare considered a key component of a collective social movement towards a better society; a joint social mission rather than a service. The socialist health care movement was characterized by a collective ethos and a grass-roots orientation, driven by slogans encouraging state officials and health care providers to stay close to the people, “eating with people, living with people, working with people” (“ăn cùng dân, ở cùng dân, làm cùng dân”). In the 1980s, however, under pressures from post-war poverty and international isolation, the socialist economic system was replaced by market-oriented reforms, and health care delivery became increasingly de-centralized, privatized and commercialized (41, 43, 44). Over the next decades, health care system attention shifted towards curative care rather than prevention, and towards tertiary rather than primary level health care, with profit-oriented health care services often being aggressively advertised and marketed (13, 45).

Notably, however, Vietnam is currently seeing a return to primary health care, with sustained government efforts to enhance health system capacities at the grassroots level, including an increased focus on patient-centered care (46, 47) – most recently with the Resolution 72 on “breakthrough solutions” to strengthen public health (48). Our ethnographic research findings point to the important potential of digital health care as a part of this return to the grassroots: digital accompaniment may be the 21^st^-century way of staying close to the people, providing health care that aligns with local lives, supporting collective mobilization for health, and nourishing a spirit of community and togetherness.

## Conclusion

Accompaniment as a key healthcare principle is currently gaining traction in global health research and programming. This article has shown how a digital self-care intervention in Vietnam became an important source of accompaniment for women with GDM. A central accomplishment of the VALID II intervention was that it accompanied women through a pregnancy journey challenged and complicated by a GDM diagnosis, providing them with a sense of direction and a feeling of being helped and supported. The intervention strengthened self-care agency, leveraged family love, and enabled reproductive empowerment through relational support.

Drawing on these empirical insights, we developed the 5-C model for digital health accompaniment. The five Cs refer to: Co-creation, Combined knowledge, Community spirit, Consistent presence, and Commitment over time. These five features emerged as vitally important general characteristics of the successful digital GDM intervention studied here, helping to create a sense of togetherness in striving for health that is critically important for advancing self-care capacities.

As discussed in this article, several elements of the 5-C model are supported by Vietnamese cultural ideals of collectivity, empathy, and mutuality, while also resonating strongly with the healthcare history of socialist Vietnam. Yet, given the importance of togetherness in human life in general, we suggest that the 5-C model has wider relevance and may be applicable in other socio-cultural settings and healthcare contexts. Future research in other settings may explore the extent to which this is the case.

For now, the present study provides important evidence that accompaniment through digital health interventions can enhance individual health and family solidarity, while also holding potential for health systems strengthening by supporting a 21^st^-century return to primary and community-oriented healthcare. Health care accompaniment is thus a significant source of empowerment for self-care that deserves further attention in research, policy, and practice.

## Data Availability

To protect the privacy of research participants, the datasets presented in this article are not publicly available. Requests to access the datasets should be directed to tine.gammeltoft@anthro.ku.dk.

## References

[1] Sheiner E . Gestational diabetes mellitus: Long-term consequences for the mother and child grand challenge: How to move on towards secondary prevention? Front Clin Diabetes Healthc. (2020) 1:546256. doi: 10.3389/fcdhc.2020.546256. PMID: 36993989 PMC10041873

[2] Ye W Luo C Huang J Li C Liu Z Liu F . Gestational diabetes mellitus and adverse pregnancy outcomes: Systematic review and meta-analysis. BMJ. (2022) 377:e067946. doi: 10.1136/bmj-2021-067946. PMID: 35613728 PMC9131781

[3] IDF (International Diabetes Federation) . IDF diabetes atlas 2025, 11^th^ edition. Brussels: International Diabetes Federation (IDF) (2025).

[4] Benton M Silverio SA Ismail K . It feels like medically promoted disordered eating: The psychosocial impact of gestational diabetes mellitus in the perinatal period. PloS One. (2023) 18:e0288395. doi: 10.1371/journal.pone.0288395. PMID: 37478148 PMC10361484

[5] OuYang H Chen B Abdulrahman A-M Li L Wu N . Associations between gestational diabetes and anxiety or depression: A systematic review. J Diabetes Res. (2021), 9959779. doi: 10.1155/2021/9959779. PMID: 34368368 PMC8337159

[6] Guernon AS . Brief illness, haunting effects: gestational diabetes and the spectrality of care Cult Med Psychiatry. (2025) 49(4):1417–1436. doi: 10.1007/s11013-025-09948-x, PMID: 41125899

[7] Craig L Sims R Glasziou P Thomas R . Women’s experiences of a diagnosis of gestational diabetes mellitus: A systematic review. BMC Pregnancy Childbirth. (2020) 20:76. doi: 10.1186/s12884-020-2745-1. PMID: 32028931 PMC7006162

[8] Pham S Churruca K Ellis LA Braithwaite J . A scoping review of gestational diabetes mellitus healthcare: Experiences of care reported by pregnant women internationally. BMC Pregnancy Childbirth. (2022) 22:627. doi: 10.1186/s12884-022-04931-5. PMID: 35941555 PMC9361509

[9] Feng YJ Deng Z Sivak A Yeung RO Nagpal T . Women’s perspectives to improve prenatal care for gestational diabetes: A systematic review and meta-aggregation of qualitative studies. Acta Obstet Gynecol Scand. (2025) 104:267–87. doi: 10.1111/aogs.14973. PMID: 39656503 PMC11782075

[10] Dang NT Le HM Nguyen A Glöde PC Vinter CA Nielsen J . Self-care interventions among women with gestational diabetes mellitus in low and middle-income countries: A scoping review. Syst Rev. (2025) 14:50. doi: 10.1186/s13643-025-02790-7. PMID: 40016820 PMC11866587

[11] Vakili F Mahmoodi Z Nasiri M Alamolhoda SH Hamzehgardeshi Z Hoseinabadi VA . Association of intermediate health determinants with gestational diabetes self-care: A structural equation modeling approach. BMC Women's Health. (2025) 25:293. doi: 10.1186/s12905-025-03844-7. PMID: 40611222 PMC12225482

[12] Nguyen DK Le HM Søndergaard Linde D Nguyen TA Søndergaard J Meyrowitsch D . Effect of an intervention during pregnancy among women with GDM in Vietnam: Clinical obstetric and neonatal outcomes Manuscript, 2026.

[13] Nguyen MP Tariq A Hinchcliff R Luu HN Dunne MP . Contribution of private health services to universal health coverage in low and middle-income countries: Factors affecting the use of private over public health services in Vietnam. Int J Health Plann Manage. (2023) 38:1613–28. doi: 10.1002/hpm.3689. PMID: 37485548

[14] Nguyen PT Gilmour S Le PM Nguyen HL Dao TMA Tran BQ . Trends in, projections of, and inequalities in non-communicable disease management indicators in Vietnam 2010–2030 and progress toward universal health coverage: A Bayesian analysis at national and sub-national levels. EClinicalMedicine. (2022) 51:101550. doi: 10.1016/j.eclinm.2022.101550. PMID: 35856038 PMC9287489

[15] Nguyen TT Trevisan M . Vietnam a country in transition: Health challenges. BMJ Nutrition Prev Health. (2020):bmjnph-2020-000069. doi: 10.1136/bmjnph-2020-000069. PMID: 33235972 PMC7664505

[16] Le HM Nguyen TA Nguyen DK Søndergaard Linde D Bygbjerg IC Søndergaard J . Prevalence and risk factors of gestational diabetes mellitus among pregnant women in northern Vietnam: A cross-sectional study. Glob Health Action. (2025) 18:2460339. doi: 10.1080/16549716.2025.2460339. PMID: 39925195 PMC11812109

[17] Nguyen TH Hoang KC . Schleiff M Bishai D , editors. Achieving health for all: primary health care in action. Johns Hopkins University Press, Baltimore (2020). doi: 10.1353/book.77991

[18] Le HTQA Nguyen MT Nguyen Vu QH Duong QT Thi LLC Thi MTN . Understanding patients’ perceptions of chronic illness care, self-management support needs and their relationship with telehealth preferences: A cross-sectional study in Vietnamese primary care. BMJ Open. (2025) 15:e090734. doi: 10.1136/bmjopen-2024-090734. PMID: 40555453 PMC12198800

[19] Nguyen TT Nguyen TT Tran BQ Pham CT Perry KE Haregu T . Putting non-communicable disease data to work in Vietnam: An investigation of community health surveillance capacity. BMC Public Health. (2023) 23:321. doi: 10.1186/s12889-023-14986-4. PMID: 36788519 PMC9926709

[20] Gammeltoft TM Nguyen TA Vu TKD Thi Dang NA Phuong Nguyen TM Nguyen VT . The pioneers of Vietnam’s epidemiological transition: An ethnographic study of pregnant women’s experiences of gestational diabetes. Global Health Action. (2024) 17. doi: 10.1080/16549716.2024.2341521. PMID: 38693861 PMC11067556

[21] WHO . WHO guideline on self-care interventions for health and well-being. Geneva: World Health Organization (2022).

[22] Burke HM Ridgeway K Murray K Mickler A Thomas R Williams K . Reproductive empowerment and contraceptive self-care: A systematic review. Sex Reprod Health Matters. (2021) 29:2090057. doi: 10.1080/26410397.2022.2090057. PMID: . Erratum in: Sex Reprod Health Matters. 2022;29(3):2124030. doi: 10.1080/26410397.2021.2124030. 35892261 PMC9336472

[23] Shohet M . Silence and sacrifice: family stories of care and the limits of love in Vietnam. Oakland: University of California Press (2021).

[24] Gammeltoft TM . Calibrating care: Family caregiving and the social weight of sympathy (tình cảm) in Vietnam. Am Anthropol. (2024) 126:596–607. doi: 10.1111/aman.13993. PMID: 41834780

[25] Farmer P Weigel JL . To repair the world: paul farmer speaks to the next generation. Berkeley: University of California Press (2013). Available online at: https://doi-org.ep.fjernadgang.kb.dk/10.1525/9780520955431 (Accessed April 1, 2026).

[26] Linde DS Le HM Vu DTK Dang NAT Nguyen AT Vu TP . A co-created self-care and informal support intervention targeting women with gestational diabetes mellitus in northern Vietnam (VALID-II): A protocol for a two-arm non-randomised feasibility study. Pilot Feasibility Stud. (2025) 11:73. doi: 10.1186/s40814-025-01657-x. PMID: 40442746 PMC12121233

[27] Burawoy M . The extended case method. Sociol Theory. (1998) 16:4–33. doi: 10.4135/9781473915480.n17. PMID: 34907622

[28] Bloom-Christen A Grunow H . What’s (in) a vignette? History, functions, and development of an elusive ethnographic sub-genre. Ethnos. (2022) 89:786–804. doi: 10.1080/00141844.2022.2052927. PMID: 41799851

[29] Xu N Han X Chen S Zhang J Gu P . Self-reported barriers in self-management of women with gestational diabetes: A systematic review of qualitative studies. Nurs Open. (2023) 10:7130–43. doi: 10.1002/nop2.1988. PMID: 37700604 PMC10563407

[30] Carrasco H Napier H Giber D Kang S Aguerreberre M Hing M . Accompanimeter 1.0: Creation and initial field testing of a tool to assess the extent to which the principles and building blocks of accompaniment are present in community health worker programs. Glob Health Action. (2019) 12:1699348. doi: 10.1080/16549716.2019.1699348. PMID: 31829114 PMC6913655

[31] Palazuelos D Farmer PE Mukherjee J . Community health and equity of outcomes: The Partners In Health experience. Lancet Global Health. (2018) 6:e491–3. doi: 10.1016/s2214-109x(18)30073-1. PMID: 29653618

[32] Jean-Baptiste MC Jean-Baptiste M Pognon PR Lutz A Mukherjee J Millien C . Matrones as accompagnateurs: A model for accompaniment. SSM Qual Res Health. (2025) 7:100541. doi: 10.1016/j.ssmqr.2025.100541. PMID: 41842036

[33] Watkins M . Psychosocial accompaniment. J Soc Political Psychol. (2015) 3:324–41. doi: 10.5964/jspp.v3i1.103. PMID: 38487599

[34] Thanh S . Y tế Việt Nam: 80 năm hành trình vì sức khỏe nhân dân (Vietnam’s Health Sector: 80 Years on a Journey for the People’s Health). Quân Đội Nhân Dân (2025). Available online at: https://www.qdnd.vn/80-nam-trien-lam-thanh-tuu-dat-nuoc-hanh-trinh-doc-lap-tu-do-hanh-phuc/y-te-viet-nam-80-nam-hanh-trinh-vi-suc-khoe-nhan-dan-842698 (Accessed April 1, 2026).

[35] Hjorth L Lupton D . Digitised caring intimacies: More-than-human intergenerational care in Japan. Int J Cult Stud. (2021) 24:584–602. doi: 10.1177/1367877920927427. PMID: 41836481

[36] Kosowicz L Tran K Khanh TT Dang TH Pham VA Ta Thi Kim H . Lessons for Vietnam on the use of digital technologies to support patient-centered care in low- and middle-income countries in the Asia-Pacific region: Scoping review. J Med Internet Res. (2023) 25:e43224. doi: 10.2196/43224. PMID: 37018013 PMC10132046

[37] Yu H Zhang G Hjorth L . Mobilizing care? WeChat for older adults’ digital kinship and informal care in Wuhan households. Mobile Media Communication. (2023) 11:294–311. doi: 10.1177/20501579221150716. PMID: 41836481

[38] Nunes ML Félix B Nunes F Santos I . Systematic development and refinement of a user-centered evidence-based digital toolkit for supporting self-care in gestational diabetes mellitus. Sci Rep. (2025) 15:12009. doi: 10.1038/s41598-025-96318-7. PMID: 40199963 PMC11978992

[39] Gammeltoft TM . Haunting images: A cultural account of selective reproduction in Vietnam. Berkeley: University of California Press (2014).

[40] Schwenkel C . Sonic socialism: crisis and care in pandemic hanoi. Oakland: University of California Press (2025).

[41] London JD . Reasserting the state in Viet Nam health care and the logics of market-Leninism. Policy Soc. (2008) 27:115–28. doi: 10.1016/j.polsoc.2008.09.005. PMID: 41842036

[42] Lincoln M . Epidemic politics in contemporary Vietnam: public health and the state. London: Bloomsbury Academic (2021).

[43] Thanh N Tran B Waye A Harstall C Lindholm L . Socialization of health care” in Vietnam: What is it and what are its pros and cons Value Health Regional Issues. (2014) 3:24–6. doi: 10.1016/j.vhri.2013.09.006. PMID: 29702932

[44] Quan NK Taylor-Robinson AW . Vietnam’s evolving healthcare system: Notable successes and significant challenges. Cureus. (2023) 15:e40414. doi: 10.7759/cureus.40414. PMID: 37456482 PMC10348075

[45] Forsberg LT . The political economy of healthcare commercialization in Vietnam. Oxford: University of Oxford, Global Economic Governance Programme (GEG) (2013).

[46] Dang TH Nguyen TA Hoang Van M Santin O Tran OMT Schofield P . Patient-centered care: Transforming the health care system in Vietnam with support of digital health technology. J Med Internet Res. (2021) 23:e24601. doi: 10.2196/24601. PMID: 34085939 PMC8214185

[47] Oanh TTM Thang NT Thu CNT Tuan KA . Viet Nam: a primary health care case study in the context of the COVID-19 pandemic. Geneva: World Health Organization (2023).

[48] Communist Party of Vietnam . Resolution of the Politbureau on some Breakthrough Solutions to Strengthen the Protection, Care and Improvement of People’s Health. Hanoi: Communist Party of Vietnam (2025). Available online at: https://thuvienphapluat.vn/van-ban/EN/The-thao-Y-te/Resolution-72-NQ-TW-2025-solutions-to-strengthen-the-protection-of-people-s-health/674262/tieng-anh.aspx (Accessed April 1, 2026).

